# Discovery of Anti-Inflammatory Triterpenoid Glucosides from the *Heritiera littoralis* Dryand

**DOI:** 10.3390/molecules28041658

**Published:** 2023-02-09

**Authors:** Xiaoqin Liang, Peng Niu, Jun Li, Xinlan Guan, Yanjun Zhang, Jian Li

**Affiliations:** 1 Guangxi Key Laboratory of Green Chemical Materials and Safety Technology, College of Petroleum and Chemical Engineering, Beibu Gulf University, Qinzhou 535000, China; 2 State Key Laboratory for the Chemistry and Molecular Engineering of Medicinal Resources, School of Chemical & Pharmaceutical Sciences, Guangxi Normal University, Guilin 541004, China; 3 Peoples’ Hospital of Pubei, Qinzhou 535300, China

**Keywords:** *Heritiera littoralis* Dryand., Heritiera A, Heritiera B, anti-inflammatory

## Abstract

Two new triterpenoid glucosides, Heritiera A (**1**) and Heritiera B (**2**), and six known triterpenoid analogs (**3**–**8**) were isolated from *Heritiera littoralis* Dryand. Their structures were identified by comprehensive spectroscopic analyses and comparisons with the literature. The anti-inflammatory activity of the isolates from *H. littoralis* was evaluated using a lipopolysaccharide (LPS) stimulated RAW 264.7 cells model. The result showed that four triterpenoids exhibited potent anti-inflammatory activity. Among these compounds, compound **2** substantially inhibits the release of nitric oxide (NO) with an IC_50_ value of 10.33 μM. The triterpenoids from *H. littoralis* could be used as potential candidates for the development of new anti-inflammatory agents.

## 1. Introduction

Inflammation is a host defense response to danger signals from pathogens, damaged cells, irradiation, etc. [1]. The characteristic signs of acute inflammation include redness, swelling, warmth, and pain, which occur during various acute infections or tissue damage [2]. In the inflammatory response, there is an increase in the permeability of endothelial lining cells and influxes of blood leukocytes into the interstitium, oxidative burst, and release of cytokines (interleukins and tumor necrosis factor-α). At the same time, there is also induction of the activity of several enzymes (oxygenases, NO synthases, and peroxidases) as well as the arachidonic acid metabolism [3]. NO is a gaseous short-lived free radical that is considered to be a mediator of inflammation. Regulating the biosynthesis or activity of NO results in the amelioration of the acute inflammation model [4]. Thus, inhibitors of some pro-inflammatory mediators from folk herbs have potential anti-inflammatory activities, which are of great interest to scientific researchers.

At present, the main drugs used in the treatment of inflammation are non-steroidal anti-inflammatory drugs, steroidal anti-inflammatory drugs, and traditional Chinese medicine. As synthetic anti-inflammatory drugs used in clinical currently have obvious adverse reactions, however traditional Chinese medicines with less toxicity and side effects have attracted more and more attention [5,6]. Natural plant components, such as triterpenoids and their glycosides, have various structural types, strong biochemical specificity, good curative effect, and low drug resistance, which are good resources for the development of potentially effective anti-inflammatory drug candidates [7,8]. These compounds have a good inhibitory effect on acute and chronic inflammation, which show an anti-inflammatory activity by inhibiting the production of inflammatory factors, and oleanane and ursane triterpenoids exhibit the most significant hypoglycemic activity, especially among triterpenoids [9,10,11].

The genus *Heritiera* belongs to the family Malvaceae growing in tropical and subtropical regions of Asia [12]. It consists of 35 species, of which 3 species can be found in the provinces of Guangdong, Guangxi, Hainan, and Yunnan in China. The genus *Heritiera* has a long history of medicinal use in China, especially for folk use [13,14]. *Heritiera littoralis* Dryand., a semi-mangrove plant, has been used as a traditional medicine for the Jing nationality [15]. This plant has been reported for its wide range of activities, including the treatment of hematuria, diarrhea, dysentery, oral infection, and toothache [16,17,18]. So far, *Heritiera littoralis* has been reported to have significant anti-inflammatory activity in the folk [14]. The isolation and characterization of the chemical constituents from *H. littoralis* have yielded a range of classes of natural products, such as triterpenoid, flavonoid, phenylpropanoid-glycerol, etc. [19,20,21,22,23]. However, there have been few reports on the biologically active constituents of *H*. *littoralis* [24].

**Figure 1:**
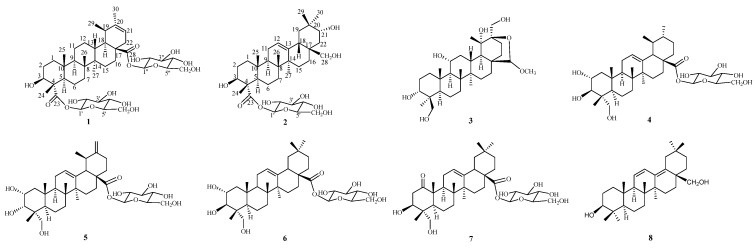
The structures of compounds (**1**)–(**8**) from *H. littoralis*.

To explore the anti-inflammatory lead compounds from the genus *Heritiera*, the active ingredients were separated from the leaves of *H*. *littoralis*. The structures of all isolated constituents were characterized by comprehensive spectroscopic analyses and comparisons with the literature. These findings have led to the isolation of two new triterpenoid glucosides, Heritiera A (**1**) and Heritiera B (**2**), and six known triterpenoid analogs (**3**–**8**) Figure 1. Furthermore, the isolates were evaluated for their anti-inflammatory activities against the production of the NO in LPS-induced inflammation in RAW264.7 macrophage cells. Herein, the isolation, purification, and determination of these isolates and the assays used to determine the production of NO in RAW264.7 macrophage cells of the constituents are described. The research provided the basis for expanding the utilization and development of this medicinal plant.

## 2. Results and Discussion

### 2.1. Elucidation of the Chemical Structure of Heritiera A (***1***) and Heritiera B (***2***)

**Figure 2:**
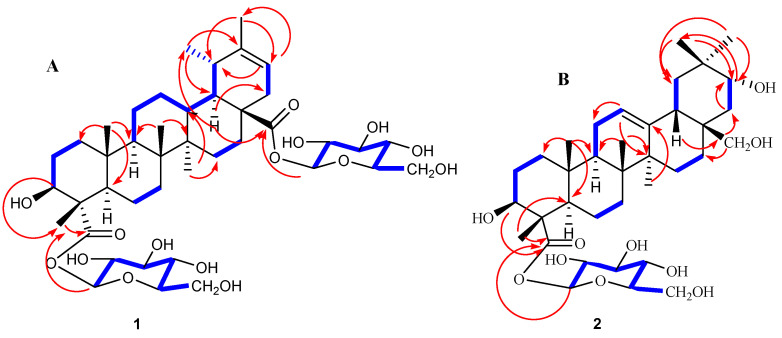
^1^H-^1^H COSY (blue bold bonds) and key HMBC (red arrows) correlations of **1** (**A**) and **2** (**B**).

**Figure 3:**
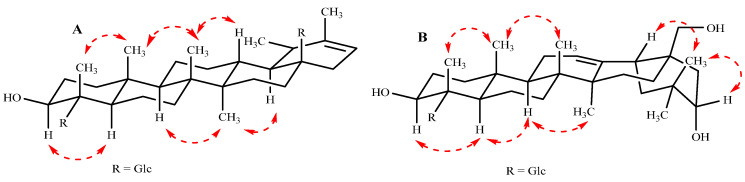
ROESY (double dashed arrows) correlations of **1** (**A**) and **2** (**B**).

Heritiera A (**1**) was obtained as a colorless gum whose molecular formula was determined to be C_42_H_66_O_15_ from the HRESIMS data at *m*/*z* 834.4338 [M + Na]^+^ with ten indices of hydrogen deficiency. The ^1^H NMR data Table 1 displayed six methyl singlets (*δ*_H_ 0.86, s, 6H; *δ*_H_ 1.05, s, 3H; *δ*_H_ 1.11, s, 3H; *δ*_H_ 1.59, s, 3H; *δ*_H_ 1.67, s, 3H), one olefinic proton (*δ*_H_ 5.35, 1H, d, *J* = 7.2 Hz), two anomeric protons *δ*_H_ 6.48 (1H, dd, *J* = 8.0, 2.8 Hz) and *δ*_H_ 6.35 (1H, dd, *J* = 8.0, 2.8 Hz), as well as overlapping signals belonging to sugar moieties around *δ*_H_ 3.88–4.39 (12 H). The ^13^C NMR and DEPT data (Table 1) revealed the existence of 42 carbon resonances, including six methyls, eleven methylenes, seventeen methines (one olefinic at *δ*_C_ 117.8), and eight quaternary carbons (two carbonyls at *δ*c 174.8, 177.8; one olefinic at *δ*_C_ 143.6). The ^1^H and ^13^C NMR spectra also showed the signals of two anomeric carbons at *δ*_H_ 6.48/*δ*_C_ 96.9 (C-1′) and *δ*_H_ 6.35/*δ*_C_ 95.7 (C-1″) indicating the presence of two sugar moieties in **1**. Furthermore, D-glucose was identified by the acid hydrolysis solution of **1** by comparison with authentic sugar samples in an HPLC assay [25]. Moreover, five of the ten indices of hydrogen deficiency were ascribed to two carboxyl groups, one double bond, and two hexacylic rings, with the remaining five indices indicating the presence of a pentacyclic system. With the aid of 2D NMR experiments, the ^1^H−^1^H COSY (Figure 2A, bold lines) and HSQC data revealed the partial structures –CH_2_ (1)−CH_2_ (2)−CH_2_ (3)−, –CH_2_ (6)−CH_2_ (7), −CH (9)−CH_2_ (11)−CH_2_ (12)−CH (13)−CH (18)−CH (19)−CH_3_ (29), –CH_2_ (15)−CH_2_ (16) and –CH (21)−CH_2_ (22)−. Detailed analyses of the abovementioned NMR data showed that the data of **1** were highly similar data to those of 3*β*-hydroxy-18α,19α-urs-20-en-28-oic acid [26,27], except for the presence of a carboxyl carbon at C-23 (*δ*_C_ 177.8 ppm) and two glycosyl units in **1**. Furthermore, the HMBC correlations from the anomeric protons H-1′ (*δ*_H_ 6.48, dd, *J* = 8.0, 2.8 Hz) to C-23 (*δ*_C_ 177.8) and H-1″ (*δ*_H_ 6.35, dd, *J* = 8.0, 2.8 Hz) to C-28 (*δ*_C_ 174.8) suggested that the two D-glucoses are situated at C-23 and C-28, respectively. The configuration of the sugar was assigned as *β* on the basis of coupling constant values (*J* = 8.0 Hz) of anomeric protons [28]. In addition, the relative stereochemistry was deduced from ROESY spectrum (double arrows in Figure 3A). In the ROESY spectrum, the correlations of H-3α/H-5α and H-9α/H-27α/H-18α indicated that H-3, H-5, H-9, H-18, and H-27 are α-oriented. The correlations of H-24*β*/H-25*β*, and H-25*β*/H-26*β*/H-13*β* suggested that H-13, Me-24, Me-25, Me-26 are *β*-oriented. Consequently, the structure of **1** was established as 3*β*-hydroxy-18α,19α-urs-20(21)-en-23,28-dioic acid-23,28-*O*-*β*-D-lucopyranosyl diester, and named Heritiera A (Figure 1).

Heritiera B (**2**) was obtained as a colorless gum whose molecular formula was determined to be C_36_H_58_O_10_ from the HRESIMS data at *m*/*z* 673.3920 [M + Na]^+^ with eight indices of hydrogen deficiency. The ^1^H NMR data (Table 1) displayed six methyl singlets (*δ*_H_ 0.92, s, 3H; *δ*_H_ 0.96, s, 3H; *δ*_H_ 1.00, s, 3H; *δ*_H_ 1.20, s, 3H; *δ*_H_ 1.34, s, 3H; *δ*_H_ 1.60, s, 3H) respectively, and one olefinic proton (*δ*_H_ 5.45, 1H, d, *J* = 8.0 Hz), one anomeric proton *δ*_H_ 6.40 (1H, d, *J* = 8.0 Hz), as well as overlapping hydrogen signals in oxygenated carbons perhaps belonging to sugar moieties. The ^13^C NMR and DEPT data (Table 1) revealed the existence of thirty-six carbon resonances, including six methyls, eleven methylenes (one olefinic at *δ*_C_ 117.6), eleven methines (one olefinic at *δ*_C_ 158.5), and eight quaternary carbons (one carbonyl at *δ*_C_ 178.1). The ^1^H and ^13^C NMR spectra also showed the signals of one anomeric carbon at *δ*_H_ 6.40 (d, *J* = 8.0 Hz)/*δ*_C_ 96.9 (C-1′), indicating the presence of one sugar moiety in the molecule, after acid hydrolysis of **2**, the D-glucose existed in **2** by analysis of HPLC [25]. In addition, the configuration of the sugar was assigned as *β* on the basis of the coupling constant value (*J* = 8.0 Hz) of the anomeric proton [28]. Three of the eight indices of hydrogen deficiency were ascribed to one carboxyl group, one double bond, and one hexacylic ring, with the remaining five indices indicating the presence of a pentacyclic system. Above mentioned facts suggested that the aglycone part of **2** was similar to that of erythrodiol [29]; the differences existed mainly in the C (23), C (21), and one more glycosyl unit in **2**. The C (23) was a carboxyl carbon with *δ*_C_ 178.1 ppm, and the C (21) was an oxygenated carbon with *δ*_C_ 73.1 ppm. Furthermore, in the HMBC spectrum (arrows in Figure 2B), the anomeric proton at *δ*_H_ 6.40 (H-1′) showed a correlation with *δ*_C_ 178.1 (C-23), which indicated that the sugar moiety is situated at C-23. In addition, the relative stereochemistry was deduced from the ROESY spectrum (double arrows in Figure 3B). In the ROESY spectrum, the correlations of H-3α/H-5α and H-9α/H-27α indicated that H-3, H-5, H-9, and H-27 are α-oriented. The correlations of H-13*β*/H-20*β,* H-24*β*/H-25*β*, and H-25*β*/H-26*β* suggested that H-13, H-20, Me-24, Me-25, Me-26 are *β*-oriented. Thus, the structure of **2** was established as 3*β*,21α,28*β*-trihydroxy-oleana-12(13)-en-23-acid-*O*-*β*-D-glucopyranosyl ester, named Heritiera B.

By comparing the measured NMR (^1^H and ^13^C) and MS data to those reported in the literature, the known triterpenoids were identified as juglansin A (**3**) [30], quadranoside IV (**4**) [31], 2α,3α,23-trihydroxy-12, 20(30)-dien-28-ursolic acid 28-*O*-*β*-D-glucopyranoside (**5**) [32], arjunglucoside II (**6**) [33], 1-oxo-3*β*,23-dihydroxy olean-12-en-28-oic acid 28-*O*-*β*-D-glucopyranoside (**7**) [34], and 3*β*,28-dihydroxy-oleana-11, 13(18) diene (**8**) [35] (Figure 1).

### 2.2. Anti-Inflammatory Assay of the Isolates

The RAW264.7 cell viability assays showed that the survival rate was greater than 90% after treatment with all isolates at different concentrations from 0 to 50 μM. The effects of all compounds on the production of NO by LPS-induced RAW 264.7 cells are shown in Table 2. Compound **2** substantially inhibited the release of NO, with an IC_50_ value of 10.33 μM. The value is slightly lower than that of the positive control, dexamethasone, with an IC_50_ value of 6.39 μM. Compounds **1**, **3**, and **4** showed moderate effects with IC_50_ values of 32.11, 39.32, and 29.98 μM, respectively. Compounds **5**, **6, 7,** and **8** showed no significant effects against LPS-induced nitric oxide production in RAW264.7 macrophages. However, the anti-inflammatory mechanism of compounds needs to be further discussed. We will conduct research in the next step.

### 2.3. Similarities and Differences of Some Triterpenoids and Their Anti-Inflammatory Activity from Malvaceae

It was reported that some triterpenoids isolated from Malvaceae exhibited good anti-inflammatory activity, such as taraxerol (oleanane triterpenoid) and lupeol (lupane triterpenoid) from *Grewia flava* roots yielded promising IC_50_ values of 21.88 ± 0.02 and 14.2 ± 0.01 μg/mL against NO production in RAW264.7 cells respectively [36]. Betulinic acid (lupane triterpenoid) and taraxasterol (ursane triterpenoid) were isolated from *Luehea ochrophylla* Mart [37]. The literature reported that betulinic acid was justified by inhibiting the release of pro-inflammatory mediators, mainly NO, IL-1*β*, TNF-α, and reduction of COX-2 levels [38,39]. Taraxasterol had efficacy comparable to prednisolone in a paw edema model induced by formalin [40]. Compound **2**, as an oleanane triterpenoid, showed anti-inflammatory activity and inhibited the release of NO, with an IC_50_ value of 10.33 μM too. These results support our initial investigation to obtain more anti-inflammatory active triterpenoids from this experiment.

## 3. Experimental Section

### 3.1. General Experimental Procedures

Optical rotations were measured on an Anton Paar MCP500 polarimeter (λ 589 nm, path length 1.0 cm). UV spectra were acquired using a TU-1901 spectrophotometer (λ 190~800 nm, slit width 2.0 nm). NMR experiments were conducted on a Bruker Advance 600 MHz or 400 MHz spectrometer with the residual solvent as an internal standard. HRESIMS were recorded by a BRUKER MAT 95XP mass spectrometer, respectively. Semi-preparative HPLC was conducted on a Waters 2545 instrument equipped with a PAD detecter and a Waters C_18_ column (5 μm, 9.3 × 250 mm) at a flow rate of 2.5 mL/min. Analytical HPLC was conducted on a Waters 2695 instrument equipped with a PAD detecter, ELSD detecter, and a Waters C_18_ column (5 μm, 4.6 × 250 mm) at a flow rate of 1.0 mL/min. OD values of 96-well were measured with an imark Bio-Rad plate microplate reader.

### 3.2. Plant Material

Dried leave (5.0 kg) of *H*. *littoralis* was obtained from Beilun Estuary in Guangxi Province, China, in September 2018 and identified by professor Qiuping Zhong (College of marine science, Beibu Gulf University). A voucher specimen (No. ID-20180910) is deposited at the Guangxi key laboratory of green chemical materials and safety technology, Beibu Gulf University, China.

### 3.3. Extraction and Isolation

Dried leaves power (5.0 kg) of *H. littoralis* was extracted with EtOH-H_2_O (20 L each, 75:25) under the extraction tank (solid-liquid ratio 1:4 g/mL, 80 °C, 4 h each, repeated 3 times), The filtrates were combined and dried under reduced pressure to yield a residue (0.52 kg, yield extract 10.4%). The concentrates were suspended in H_2_O (1 L) and partitioned with EtOAc (5 × 1 L) and n-BuOH fraction (5 × 1 L). The n-BuOH fraction (125.3 g) was subjected to silica gel CC (200~300 mesh) and eluted sequentially with CHCl_3_-MeOH (100:0, 90:10, 80:20, 50:50, and 0:100, *v*/*v*) to yield five fractions (A–E). Fraction B (13.2 g) was using RP-C_18_ silica gel CC and eluted with H_2_O-MeOH (40:60, 30:70, 20:80, 0:100, *v*/*v*) to afford four subfractions; further separation of subfraction B-2 using Sephadex LH-20 CC (eluted with 100% MeOH) yielded compounds **8** (8.9 mg). Fraction C (35.2 g) was purified using RP-C_18_ silica gel CC and eluted with H_2_O-MeOH (70:30, 60:40, 50:50, 40:60, 30:70, 20:80, 10:90, 0:100, *v*/*v*) to afford eight subfractions (C-1~C-8). Fraction C-1 (1.1 g) was fractionated by silica gel CC with a CH_2_Cl_2_-EA gradient elution (100:0 → 0:100, *v*/*v*) to afford five fractions C-1-(1–5). Fraction C-1-2 was subjected to Sephadex LH-20 CC (eluted with 100% MeOH) and recrystallization to yield compounds **5** (5.6 mg) and **7** (4.9 mg). Fraction C-1-3 was subjected to Sephadex LH-20 CC (eluted with H_2_O-MeOH (50:50 → 0:100), Subfraction C-1-3-2 was separated using semi-preparative HPLC (9.3 × 250 mm, 5 μm, SB-C_18_, 2.5 mL/min, λ = 230 nm) eluted with H_2_O-MeOH (40:60) to obtain compounds **4** (t_R_ 21.4 min, 7.0 mg) and **6** (t_R_ 35.9 min, 6.2 mg). Subfraction C-2-3 (231.3 mg) was separated using semi-preparative HPLC (9.3 × 250 mm, 5 μm, SB-C_18_, 2.5 mL/min), eluted with H_2_O-MeOH (55:45 → 25:75) to afford six subfractions C-2-3-1 to C-2-3-6. Compounds **1** (t_R_ 23.4 min, 3.0 mg), **3** (t_R_ 35.4 min, 2.5 mg), and **2** (t_R_ 43.2 min, 3.6 mg) were obtained from subfractions C-2-3-3 using semi-preparative HPLC (9.3 × 250 mm, 5 μm, SB-C_18_, 2.5 mL/min, λ = 210 nm) eluted with H_2_O-CH_3_CN, 35:65). (Figure S20 Supplementary Materials). The yield was calculated as follows:

Yield extract % = weight of dry extract/weight of dried plant material × 100%(1)

### 3.4. Characterization of the Isolates

Heritiera A (**1**): Colorless gum; [α] ^25^_D_ +15.7 (*c* 0.2, MeOH); HRESIMS *m*/*z* 833.4304 [M + Na]^+^, calcd for C_42_H_66_O_15_Na). For ^1^H (pyridine-*d*_5_, 600 MHz) and ^13^C NMR (pyridine-*d*_5_, 150 MHz) spectroscopic data, see Table 1; The spectral data identified Heritiera A (**1)** as a new compound. All significant data are given in the electronic supporting information materials (Figures S1−S8).

Heritiera B (**2**): Colorless gum; [α] ^25^_D_ + 13.5 (*c* 0.20, MeOH); HRESIMS *m*/*z* 673.3920 ([M + Na]^+^, calcd for C_36_H_58_O_10_Na). For ^1^H (pyridine-*d*_5_, 600 MHz) and ^13^C NMR (pyridine-*d*_5_, 150 MHz) spectroscopic data, see Table 1; All significant data are given in the online Supplementary Material (Figures S9−S16).

Juglansin A (**3**): Colorless amorphous power. ^1^H NMR (pyridine-*d*_5_, 600 MHz) *δ*_H_ 4.51 (1H, brs, H-3), 4.41–4.36 (3H, m, H-11, 28, 30a), 4.24 (1H, d, *J* = 10.7 Hz, H-24a), 4.12 (1H, d, *J* = 11.1 Hz, H-30b), 3.97 (1H, d, *J* = 10.8 Hz, H-24b), 3.41 (3H, s, -OCH_3_), 1.85 (3H, s, H-29), 1.69 (3H, s, H-23), 1.41 (3H, s, H-25), 1.17 (3H, s, H-26), 0.96 (3H, s, H-27). ^13^C NMR (pyridine-*d*_5_, 150 MHz) *δ*_C_ 37.1 (C-1), 27.4 (C-2), 70.4 (C-3), 44.9 (C-4), 50.7 (C-5), 19.3 (C-6), 36.2 (C-7), 43.6 (C-8), 57.7 (C-9), 40.3 (C-10), 70.1 (C-11), 42.2 (C-12), 41.6 (C-14), 28.5 (C-15), 31.7 (C-16), 34.7 (C-17), 50.1 (C-18), 76.6 (C-19), 78.1 (C-20), 23.6 (C-21), 35.7 (C-22), 24.5 (C-23), 66.3 (C-24), 18.2 (C-25), 17.6 (C-26), 15.9 (C-27), 109.8 (C-28), 19.2 (C-29), 65.9 (C-30), 54.9 (C-OCH_3_).

Quadranoside IV (**4**): Colorless amorphous power. ^1^H NMR (pyridine-*d*_5_, 600 MHz) *δ*_H_ 5.45 (1H, br s, H-12), 1.22 (3H, s, H-26), 1.12 (3H, s, H-25), 1.10 (3H, s, H-27), 1.08 (3H, s, H-24), 0.92 (3H, d, *J* = 6.1 Hz, H-29), 0.88 (3H. br s, H-30); sugar signals: *δ*_H_ 6.31 (1H, d, *J* = 8.0 Hz, H-1′), 4.35–4.05 (4H, m, H-2′, 3′, 4′ 5′), 4.44 (2H, m, H-6′). ^13^C NMR (pyridine-*d*_5_, 100 MHz) *δ*_C_ 48.5 (C-1), 69.3 (C-2), 78.5 (C-3), 44.1 (C-4), 48.5 (C-5), 18.8 (C-6), 33.5 (C-7), 40.6 (C-8), 48.2 (C-9), 38.7 (C-10), 24.1 (C-11), 126.4 (C-12), 138.8 (C-13), 42.9 (C-14), 29.0 (C-15), 25.0 (C-16), 48.7 (C-17), 53.6 (C-18), 39.6 (C-19), 39.5 (C-20), 31.1 (C-21), 37.1 (C-22), 66.7 (C-23), 14.8 (C-24), 18.0 (C-25), 18.1 (C-26), 24.2 (C-27),176.6 (C-28), 17.7 (C-29), 21.6 (C-30), 96.1 (C-1′), 74.4 (C-2′), 79.3 (C-3′), 71.5 (C-4′), 79.6 (C-5′), 62.6 (C-6′).

2α,3α,23-trihydroxy-12, 20(30)-dien-28-ursolic acid 28-*O*-*β*-glucopyranoside (**5**): Colorless amorphous power. ^1^H NMR (MeOD, 400 MHz) *δ*_H_ 5.31 (1H, t, *J* = 3.7 Hz, H-12), 4.70 (1H, brs, H-30a), 4.66 (1H, brs, H-30b), 3.81 (1H, dd, *J* = 11.9, 1.8 Hz, H-23a), 3.69 (1H, dd, *J* = 11.9, 4.4 Hz, H-23b), 1.21 (s, 3H, H-26), 1.05 (3H, s, H-27), 1.03 (3H, s, H-25), 0.86 (3H, s, H-29), 0.80 (3H, s, H-24); sugar signals: *δ*_H_ 5.36 (1H, d, *J* = 8.0 Hz, H-1′), 3.55–3.35 (4H, m, H-2′, 3′, 4′, 5′), 3.89 (1H, m, H-6′a), 3.63 (1H, m, H-6′b). ^13^C NMR (MeOD, 100 MHz) *δ*_H_ 43.5 (C-1), 67.2 (C-2), 78.3 (C-3), 42.4 (C-4), 44.2 (C-5), 19.0 (C-6), 33.7 (C-7), 41.0 (C-8), 49.3 (C-9), 39.1 (C-10), 24.5 (C-11), 127.4 (C-12), 139.0 (C-13), 42.5 (C-14), 29.2 (C-15), 25.2 (C-16), 49.6 (C-17), 56.4 (C-18), 38.5 (C-19), 154.3 (C-20), 33.2 (C-21), 39.7 (C-22), 71.1 (C-23), 17.9 (C-24), 17.5 (C-25), 17.6 (C-26), 24.0 (C-27), 177.2 (C-28), 16.7 (C-29), 105.4 (C-30), 95.7 (C-1′), 73.9 (C-2′), 78.6 (C-3′), 71.3 (C-4′), 78.7 (C-5′), 62.4 (C-6′).

Arjunglucoside II (**6**): Colorless amorphous power. ^1^H NMR (pyridine-*d*_5_, 400 MHz) *δ*_H_ 5.43 (1H, brs, H-12), 4.31 (1H, m, H-3), 4.24 (1H, m, H-2), 1.17 (3H, s, H-26), 1.16 (3H, s, H-27), 1.11 (3H, s, H-25), 1.08 (3H, s, H-24), 0.87 (6H, s, H-29, 30); sugar signals: δ_H_ 6.37 (1H, d, *J* = 8.0 Hz, H-1′), 4.24–4.46 (4H, m, H-2′, H-3′, H-4′, H-5′), 3.73 (1H, d, *J* = 10.2 Hz, H-6′a), 3.20 (1H, d, *J* = 10.2 Hz, H-6′b). ^13^C NMR (pyridine-*d*_5_, 100 MHz) *δ*_H_ 48.2 (C-1), 69.2 (C-2), 78.5 (C-3), 44.0 (C-4), 48.6 (C-5), 18.9 (C-6), 33.1 (C-7), 40.4 (C-8), 48.2 (C-9), 38.8 (C-10), 24.3 (C-11), 123.3 (C-12), 144.5 (C-13), 42.0 (C-14), 28.6 (C-15), 23.7 (C-16), 47.3 (C-17), 42.5 (C-18), 46.4 (C-19), 31.1 (C-20), 34.3 (C-21), 32.8 (C-22), 66.7 (C-23), 14.8 (C-24), 17.8 (C-25), 18.0 (C-26), 26.4 (C-27), 176.8 (C-28), 33.4 (C-29), 24.0 (C-30), 96.1 (C-1′), 74.5 (C-2′), 79.3 (C-3′), 71.4 (C-4′), 79.8 (C-5′), 62.5 (C-6′).

1-oxo-3*β*,23-dihydroxy olean-12-en-28-oic acid 28-*O*-*β*-D-glucopyranoside (**7**): Colorless amorphous power. ^1^H NMR (MeOD, 600 MHz) *δ*_H_ 5.25 (1H, t, *J* = 3.5 Hz, H-12), 3.78 (1H, m, H-3), 1.18 (3H, s, H-27), 0.94 (3H, s, H-30), 0.92 (3H, s, H-29), 0.87 (6H, s, H-24, 26); sugar signals: *δ*_H_ 5.38 (1H, d, *J* = 8.2 Hz, H-1′), 3.38–3.64 (6H, m, H-2′, H-3′, H-4′, H-5′, H-6′). ^13^C NMR (MeOD, 150 MHz) *δ*_C_ 215.2 (C-1), 44.7 (C-2), 73.4 (C-3), 44.1 (C-4), 47.7 (C-5), 18.5 (C-6), 33.3 (C-7), 40.5 (C-8), 40.3 (C-9), 53.3 (C-10), 26.3 (C-11), 124.2 (C-12), 144.2 (C-13), 43.3 (C-14), 28.9 (C-15), 24.0 (C-16), 48.2 (C-17), 42.8 (C-18), 47.0 (C-19), 31.5 (C-20), 34.9 (C-21), 33.5 (C-22), 65.8 (C-23), 13.3 (C-24), 16.0 (C-25), 18.4 (C-26), 26.2 (C-27), 178.1 (C-28), 33.1 (C-29), 23.9 (C-30), 95.7 (C-1′), 78.7 (C-5′), 78.3 (C-3′), 73.9 (C-2′), 71.1 (C-4′), 62.4 (C-6′).

3*β*,28-dihydroxyoleana-11, 13(18) diene (**8**): 3*β*,28-dihydroxy-oleana-11, 13(18) diene (8): Colorless amorphous power. ^1^H NMR (CDCl_3_, 400 MHz) *δ*_H_ 6.46 (1H, dd, *J* = 10.6, 1.8 Hz, H-12), 5.61 (1H, dd, *J* = 10.8, 1.2 Hz, H-11), 3.20 (1H, dd, *J* = 9.4, 6.8 Hz, H-3), 1.00 (3H, s, H-27), 0.98 (3H, s, H-23), 0.97 (3H, s, H-30), 0.90 (3H, s, H-25), 0.80 (3H, s, H-29), 0.78 (3H, s, H-24), 0.73 (3H, s, H-26). ^13^C NMR (CDCl_3_, 100 MHz) *δ*_C_ 38.2 (C-1), 32.8 (C-2), 79.1 (C-3), 39.0 (C-4), 54.9 (C-5), 18.5 (C-6), 32.5 (C-7), 36.8 (C-8), 54.3 (C-9), 40.4 (C-10), 126.6 (C-11), 125.4 (C-12), 137.4 (C-13), 42.2 (C-14), 24.6 (C-15), 30.5 (C-16), 39.8 (C-17), 133.8 (C-18), 38.1 (C-19), 33.1 (C-20), 35.2 (C-21), 27.2 (C-22), 28.0 (C-23), 15.2 (C-24), 18.2 (C-25), 16.6 (C-26), 20.5 (C-27), 65.4 (C-28), 24.4 (C-29), 32.4 (C-30).

### 3.5. Enzymatic Hydrolysis of Compounds ***1**–**2***

Acidic hydrolysis of compounds **1**–**2** was carried out according to the method described previously [25]. The configuration of the sugar moiety was determined by comparing the R_f_ values with the derivatives of authentic samples. The R_f_ values were 0.36 (D-glucose). D-glucose was confirmed by comparison of their retention times and optical rotations with those of authentic samples t_R_ (CH_3_CN:H_2_O, 78:22, *v*/*v*, 1 mL/min): 12.3 min (D-glucose, positive optical rotation).

### 3.6. Cell Viability and Anti-Inflammatory Activity Test [41,42]

All terpenoids isolated from the leaves of *H. littoralis* were subjected to MTT assays to assess the cell viability of LPS-stimulated RAW264.7 cell models.

Cell viability: The cell culture was maintained on RPMI-1640 medium supplemented with 10% fetal bovine serum, 100 U/mL penicillin, and 100 μg/mL streptomycin in 25 cm^2^ culture flasks at 37 °C humidified atmospheres with 5% CO_2_. All the cells to be tested in the following assays have a passage number of 3–6. The RAW264.7 macrophage cells (5.0×10^3^) per well were seeded in triplicate in 96-well plates and incubated for 24 h at 37 °C and 5% CO_2_/95% air. Then the cells were incubated for 12 h before treatment to reach 70% confluency, and the cells were stimulated with 10 μL 2 μg/mL LPS; after 2 h, 20 µL of tested various concentrations of compounds or dexamethasone (concentration of compounds ranges from 0 to 50 μM) were added to each well. The final concentration of Heritiera A (**1**), Heritiera B (**2**), juglansin A (**3**), quadranoside IV (**4**), 2α,3α,23-trihydroxy-12, 20(30)-dien-28-ursolic acid 28-O-*β*-D-gluco pyranoside (**5**), arjunglucoside II (**6**), 1-oxo-3*β*,23-dihydroxy olean-12-en-28-oic acid 28-O-*β*-D-glucopyranoside (**7**), and 3*β*,28-dihydroxy-oleana-11, 13(18) diene (**8**) were kept at 6.25, 12.5, 2.5.0, 50.0 µM, respectively. After 24 h of culture, 0.1 mg of MTT (in 20 μL of PBS) was added to each well, and cells were incubated at 37 °C for 6 h. The formed formazan°Crystals were then dissolved in 100 μL of DMSO, and the absorbance was read by enzyme labeling instrument with 570 nm wavelength measurement. Untreated PBMC was used as the unstimulated cell culture control.

Anti-inflammatory activity test: The anti-inflammatory activity of the isolates was evaluated by production of NO in RAW 264.7 cells by the Griess assay. Incubated procedure of cells was same as cell viability assay, a series of compounds treated with cell after 24 h, then production of NO in cell supernatant was determined based on the Griess reaction, and the absorbance was measured at 550 nm in a microplate reader. The final IC_50_ values were calculated (*n* = 5). The IC_50_ values are presented as the mean ± SD (standard deviation of the average value) from five independent experiments.

## 4. Conclusions

To explore the anti-inflammatory lead compounds of *H*. *littoralis* (Malvaceae), a phytochemical investigation of n-BuOH extract from the leaves of *H*. *littoralis* was carried out. Two new compounds, Heritiera A (**1**) and Heritiera B (**2**), and six known triterpenoids (**3–8**) were isolated from the leaves of *H. littoralis*. Four triterpenoids decreased the production of NO on LPS-stimulated RAW 264.7 cells. Among these compounds, compound **2** substantially inhibits the release of NO, with an IC_50_ value of 10.33 μM. Compounds **1**, **3**, and **4** showed moderate effects with IC_50_ values of 32.11, 39.32, and 29.98 μM, respectively. Collectively, *H. littoralis* leaves contain abundant triterpenoids that affect the production of NO in RAW 264.7 cells, which could be meaningful for the development of new anti-inflammatory agents.

## Figures and Tables

**Table 1:** ^1^H NMR (600 MHz), ^13^C NMR (150 MHz) data for Heritiera A (**1**) and Heritiera B (**2**). (in Pyridine-*d*_5_).

	1		2	
Positition	*δ*_H_ (*J* = Hz)	*δ*_C_ (DEPT)	*δ*_H_ (*J* = Hz)	*δ*_C_ (DEPT)
1	1.70, m; 1.20, m	39.7, CH_2_	1.54, m	38.5, CH_2_
2	1.96, m; 1.72, m	28.2, CH_2_	1.89, m	27.8, CH_2_
3	4.71, m	75.7, CH	4.64, dd, (10.6, 5.9)	75.5, CH
4		55.3, C		55.5, C
5	1.88, m	52.8, CH	1.90, m	52.4, CH
6	1.48, m	21.9, CH_2_	1.48, m; 1.65, m	18.3, CH_2_
7	1.52, m; 1.26 m	22.3, CH_2_	1.72 m; 1.64, m	22.1, CH_2_
8		42.6, C		40.1, C
9	1.46, m	51.4, CH	1.49, m	49.8, CH
10		37.3, C		38.0, C
11	1.74, m	34.7, CH_2_	2.61, dd, (14.8, 8.1); 1.88, m	31.6, CH_2_
12	1.95, m; 1.16, m	28.2, CH_2_	5.45, d, (8.0)	117.6, CH
13	2.67, m	39.8, CH		158.5, C
14		42.1, C		38.1, C
15	2.00, m; 1.04, m	29.8, CH_2_	1.47, m; 1.58, m	34.5, CH_2_
16 17 18 19 20 21 22 23 24 25 26 27 28 29 30 1′ 2′ 3′ 4′ 5′ 6′ 1″ 2″ 3″ 4″ 5″ 6′	2.44 m 1.29, m 2.55, d, (12.5) 5.35, d, (7.2) 2.40, m; 2.03, m 1.59, s 0.86, s 1.11, s 0.86, s 1.05, s 1.67, s 6.50, d, (8.2) 4.25, m 4.25, m 4.38, m 3.88, dd, (6.6, 3.2) 4.39, m 6.40, d, (8.2) 4.14, m 4.32, m 4.38, m 4.05, m 4.39, m	33.7, CH_2_ 49.8, C 49.7, CH 38.1, CH 143.6, C 117.8, CH 38.0, CH_2_ 177.8, C 12.2, CH_3_ 17.5, CH_3_ 16.6, CH_3_ 15.5, CH_3_ 174.8, C 24.0, CH_3_ 22.7, CH_3_ 96.9, CH 74.9, CH 79.4, CH 71.5, CH 79.7, CH 62.6, CH_2_ 95.7, CH 74.9, CH 79.3, CH 71.4, CH 80.0, CH 62.6, CH_2_	1.43, m; 1.80, m 0.75, dd, (13.4, 3.7) 1.81, m; 1.25, m 4.15, dd, (12.5, 3.5) 1.65, m; 2.51, m 1.60, s 0.92, s 1.00, s 0.96, s 3.63, m; 3.49, m 1.20, s 1.34, s 6.40, d, (8.0) 4.23, m 4.28, m 4.29, m 4.04, br s 4.40, d, (6.5); 4.32, dd, (11.8, 4.6)	41.6, CH_2_ 42.8, C 45.3, CH 38.7, CH_2_ 35.1, C 73.1, CH 38.5, CH_2_ 178.1, C 12.5, CH_3_ 16.5, CH_3_ 26.8, CH_3_ 22.7, CH_3_ 65.5, CH_2_ 29.2, CH_3_ 26.3, CH_3_ 96.9, CH 74.7, CH 78.9, CH 71.5, CH 79.8, CH 62.5, CH_2_

**Table 2:** Anti-inflammatory effects of compounds **1–8** on the production of the NO in LPS-stimulated RAW264.7 Cells ^a^.

Compounds	IC_50_ (μM)
**1**	32.11 ± 0.62
**2**	10.33 ± 0.43
**3**	39.32 ± 0.75
**4**	29.98 ± 0.42
**5**	>50
**6**	>50
**7**	>50
**8**	>50
Dexamethasone ^b^	6.39 ± 0.64

^a^ All values are means of three independent experiments. Values present mean ± SD of triplicate experiments. ^b^ Dexamethasone as positive control.

## Data Availability

The data of the article can be obtained from the authors.

## Supplementary Materials

The following supporting information can be downloaded at: https://www.mdpi.com/article/10.3390/molecules28041658/s1, Figure S1: HRESIMS spectrum of compound **1**; Figure S2: ^1^H NMR (600 MHz, Pyridine-*d*_5_) spectrum of compound **1**; Figure S3: ^13^C NMR (125 MHz, Pyridine-*d*_5_) spectrum of compound **1**; Figure S4: DEPT (125 MHz, Pyridine-*d*_5_) spectrum of compound **1**; Figure S5: ^1^H-^1^H COSY (600 MHz, Pyridine-*d*_5_) spectrum of compound **1**; Figure S6: HSQC spectrum of compound **1**; Figure S7: HMBC spectrum of compound **1**; Figure S8: ROESY spectrum of compound **1**; Figure S9: HRESIMS spectrum of compound **2**; Figure S10: ^1^H NMR (600 MHz, Pyridine-*d*_5_) spectrum of compound **2**; Figure S11: ^13^C NMR (125 MHz, Pyridine-*d*_5_) spectrum of compound **2**; Figure S12: DEPT (125 MHz, Pyridine-*d*_5_) spectrum of compound **2**; Figure S13: ^1^H-^1^H COSY (600 MHz, Pyridine-*d*_5_) spectrum of compound **2;** Figure S14: HSQC spectrum of compound **2**; Figure S15: HMBC spectrum of compound **2**; Figure S16: ROESY spectrum of compound **2**; Figure S17: The structures of compounds **1**–**8** from *H. littoralis*; Figure S18: ^1^H-^1^H COSY (blue bold bonds) and key HMBC (red arrows) correlations of **1**(A) and **2**(B); Figure S19: ROESY (double dashed arrows) correlations of **1**(A) and **2**(B); Figure S20: The flowchart for the isolation procedure of *H. littoralis.*
