# Supplementary material for: Discovery of Anti-Inflammatory Triterpenoid Glucosides from the Heritiera littoralis Dryand
Source: Molecules. 2023 Feb 9;28(4):1658. doi: 10.3390/molecules28041658 (PMC9959541; doi:10.3390/molecules28041658)
Supplement: Supplementary file 1 [file molecules-28-01658-s001.zip › molecules-2138096-supplementary.pdf]

SUPPLEMENTARY MATERIAL

Discovery of Anti-inflammatory Triterpenoid Glucosides from the *Heritiera littoralis* Dryand.

Xiao qin Liang [2], Peng Liu[1], Jun Li [2], Xinlan Guan [3], Yanjun Zhang [1*], Jian Li [3**]

[1] Guangxi Key Laboratory of Green Chemical Materials and Safety Technology，College of Petroleum and Chemical Engineering, Beibu Gulf University, (Qinzhou 535000)

[2] State Key Laboratory for the Chemistry and Molecular Engineering of Medicinal Resources, School of Chemical & Pharmaceutical Sciences, Guangxi Normal University, Guilin 541004, China

[3] Peoples' Hospital of Pubei, Pubei, 535300, China

* Correspondence: zhangyj201608@163.com, Tel: 086-0777-2696809; Lj877138@163.com, Tel: 086-0777-8215818.

Abstract: Two new triterpenoid glucosides, heritiera A (**1**) and heritiera B (**2**), and six known triterpenoid analogs (**3**-**8**) were isolated from *Heritiera littorali*s Dryand.. Their structures were identified by comprehensive spectroscopic analyses and comparison with literature. Anti-inflammatory activity of the isolates from *H. littorali*s was evaluated using lipopolysaccharide (LPS) stimulated RAW 264.7 cells model. The result showed that four triterpenoids exhibited potent anti-inflammatory activity. Among these compounds, coupound **2** substantially inhibit the release of nitric oxide (NO) with IC_{50} value of 10.33 μM. The triterpenoids from *H. littorali*s could be used as potential candidates for the development of new anti-inflammatory agents.

Keywords: *Heritiera littoralis* Dryand.; heritiera A; heritiera B; anti-inflammatory

List of supporting information

Figure S1. HRESIMS spectrum of compound **1** 1
Figure S2. ^{1}H NMR (600 MHz, Pyridine-d_5) spectrum of compound **1** 1
Figure S3. ^{13}C NMR (125 MHz, Pyridine-d_5) spectrum of compound **1** 2
Figure S4. DEPT (125 MHz, Pyridine-d_5) spectrum of compound **1** 2
Figure S5. ^{1}H-^{1}H COSY (600 MHz, Pyridine-d_5) spectrum of compound **1** 3
Figure S6. HSQC spectrum of compound **1** 3
Figure S7. HMBC spectrum of compound **1** 4
Figure S8. ROESY spectrum of compound **1** 4
Figure S9. HRESIMS spectrum of compound **2** 5
Figure S10. ^{1}H NMR (600 MHz, Pyridine-d_5) spectrum of compound **2** 5
Figure S11. ^{13}C NMR (125 MHz, Pyridine-d_5) spectrum of compound **2** 6
Figure S12. DEPT (125 MHz, Pyridine-d_5) spectrum of compound **2** 6
Figure S13. ^{1}H-^{1}H COSY (600 MHz, Pyridine-d_5) spectrum of compound **2** 7
Figure S14. HSQC spectrum of compound **2** 7
Figure S15. HMBC spectrum of compound **2** 8
Figure S16. ROESY spectrum of compound **2** 8
Figure S17. The structures of compounds **1-8** from *H. littoralis* 9
Figure S18. ^{1}H-^{1}H COSY (blue bold bonds) and key HMBC (red arrows) correlations of **1** (A) and **2** (B) 9
Figure S19. ROESY (double dashed arrows) correlations of **1** (A) and **2** (B) 9
Figure S20 The flowchart for the isolation procedure of *H. littoralis* 10

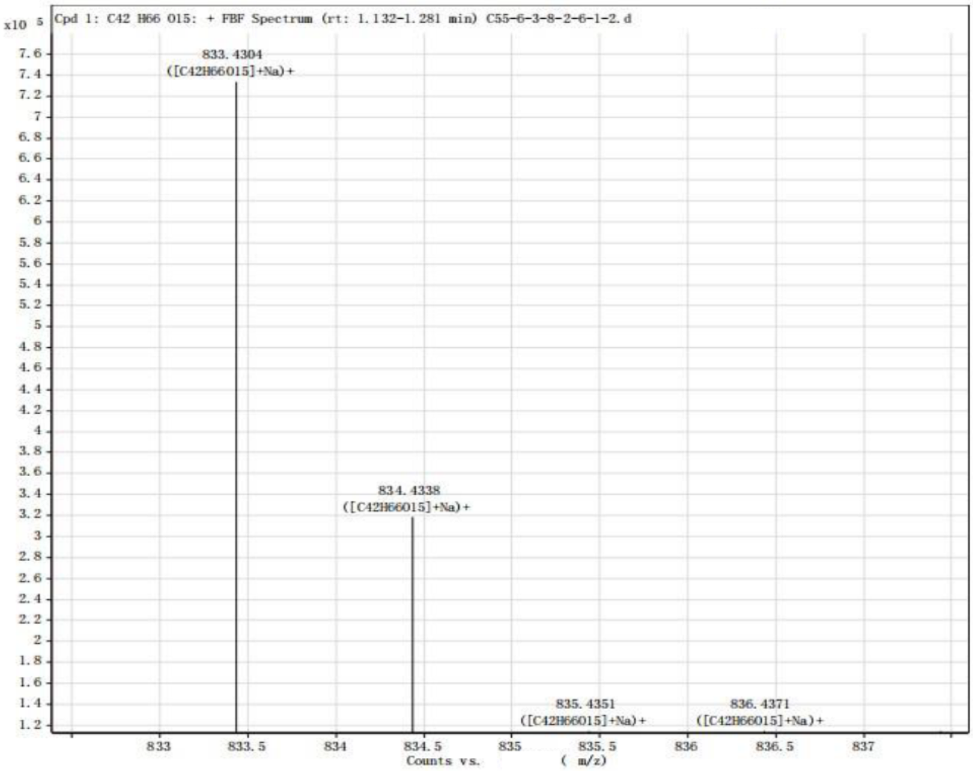

Figure S1. HRESIMS spectrum of compound **1.**

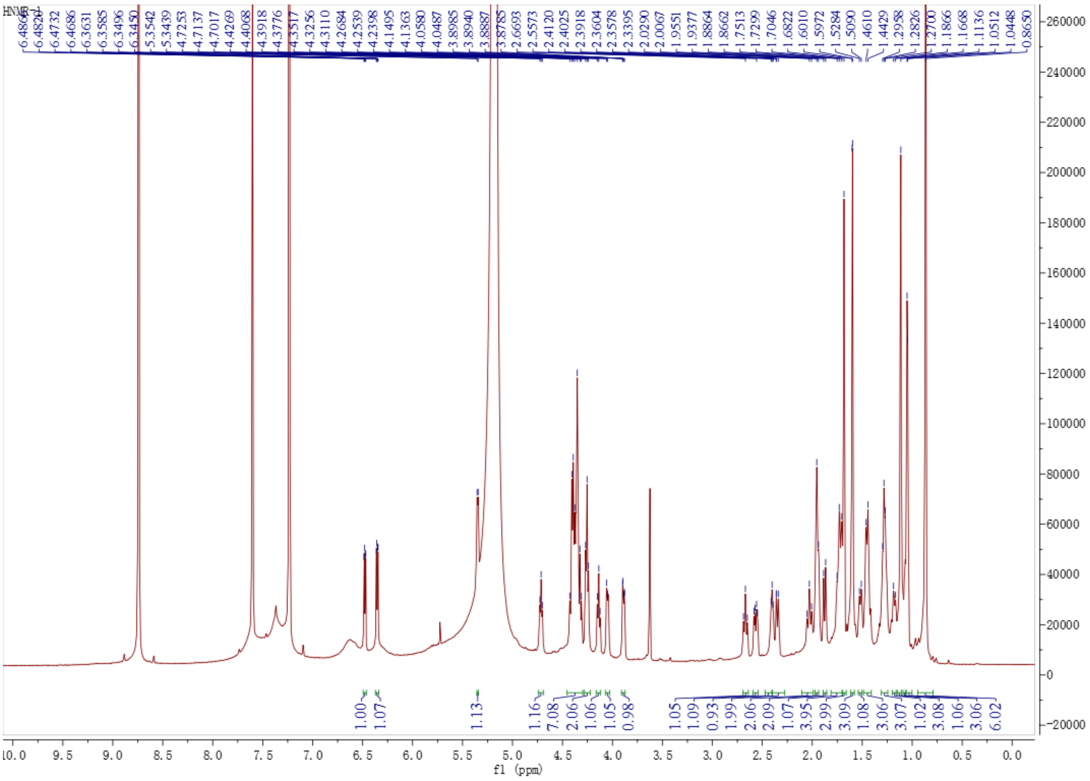

Figure S2. ^{1}H NMR (600 MHz, Pyridine-d_5) spectrum of compound **1.**

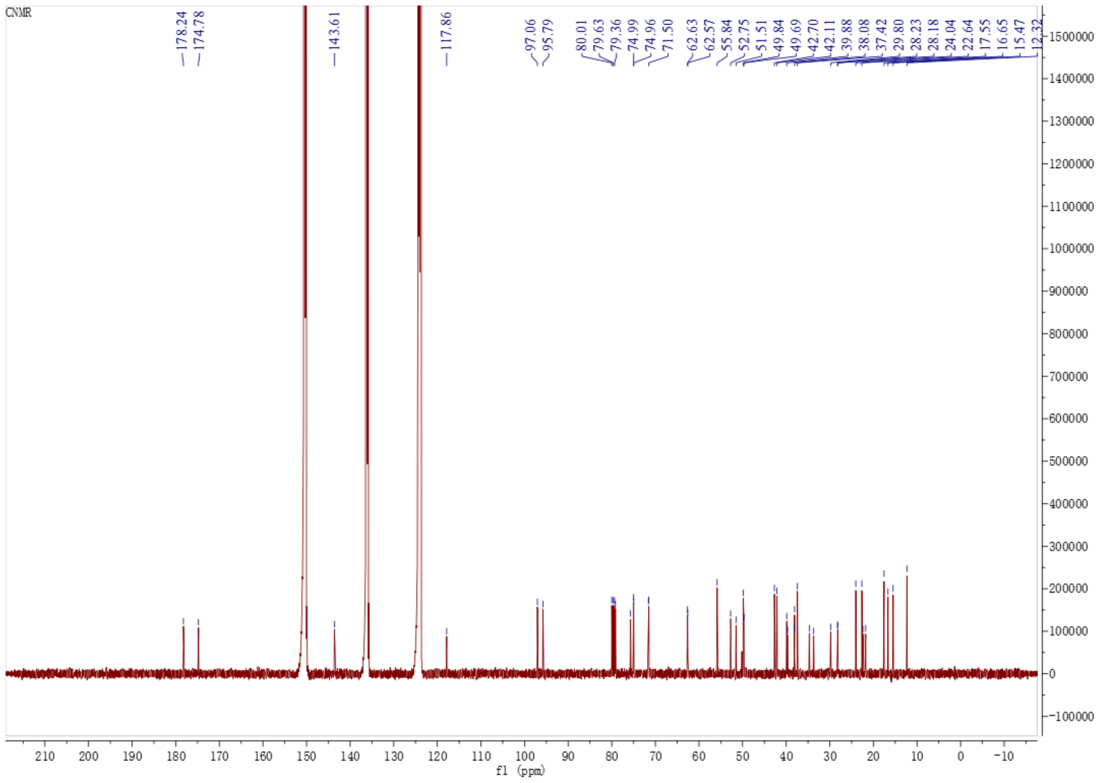

Figure S3. ^{13}C NMR (125 MHz, Pyridine-d_5) spectrum of compound **1**.

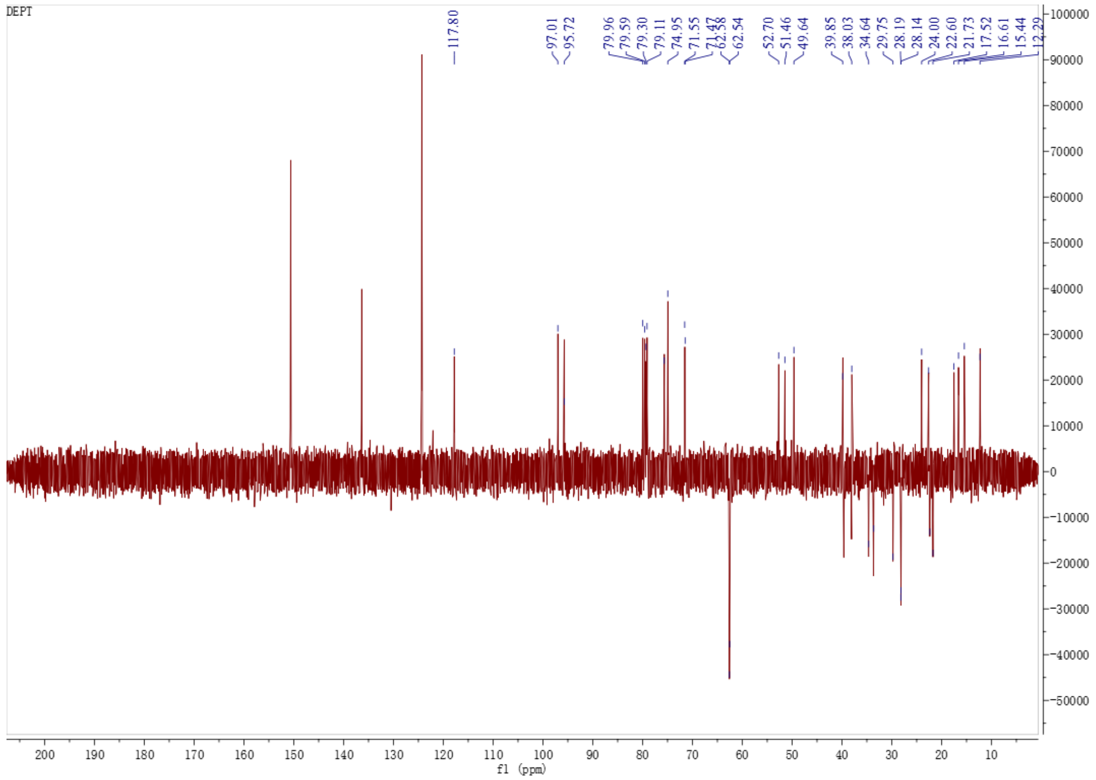

Figure S4. DEPT (125 MHz, Pyridine-d_5) spectrum of compound **1.**

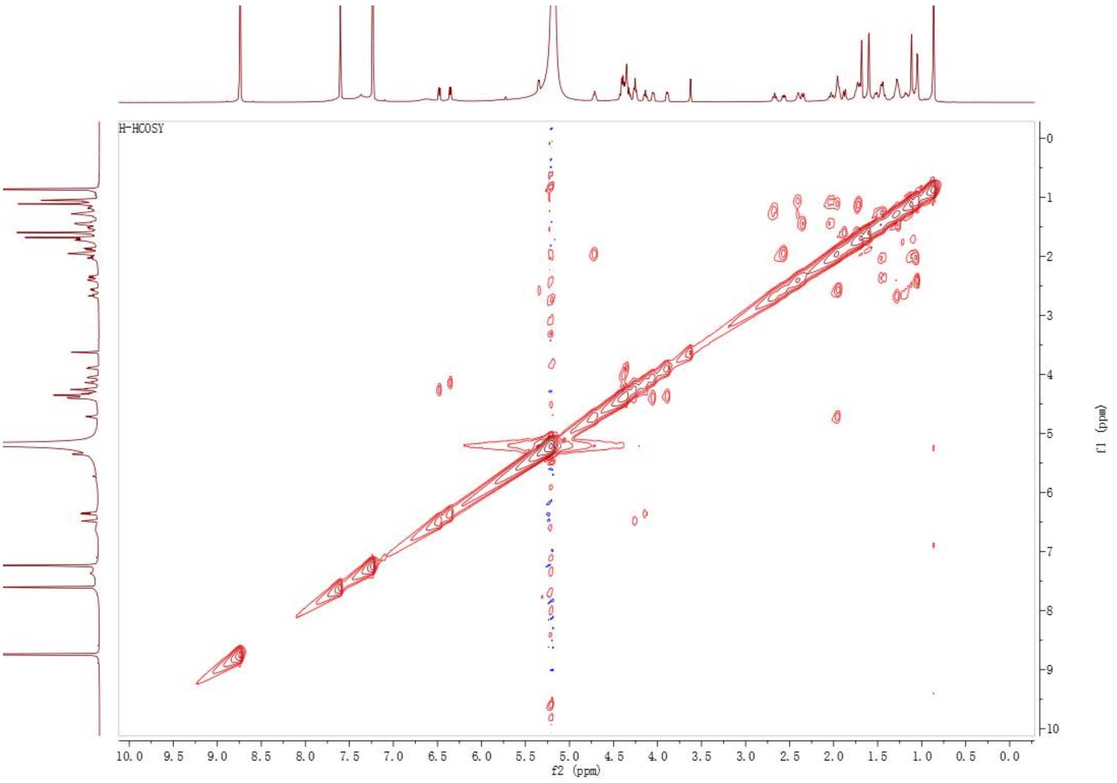

Figure S5. ^{1}H-^{1}H COSY (600 MHz, Pyridine-d_5) spectrum of compound **1.**

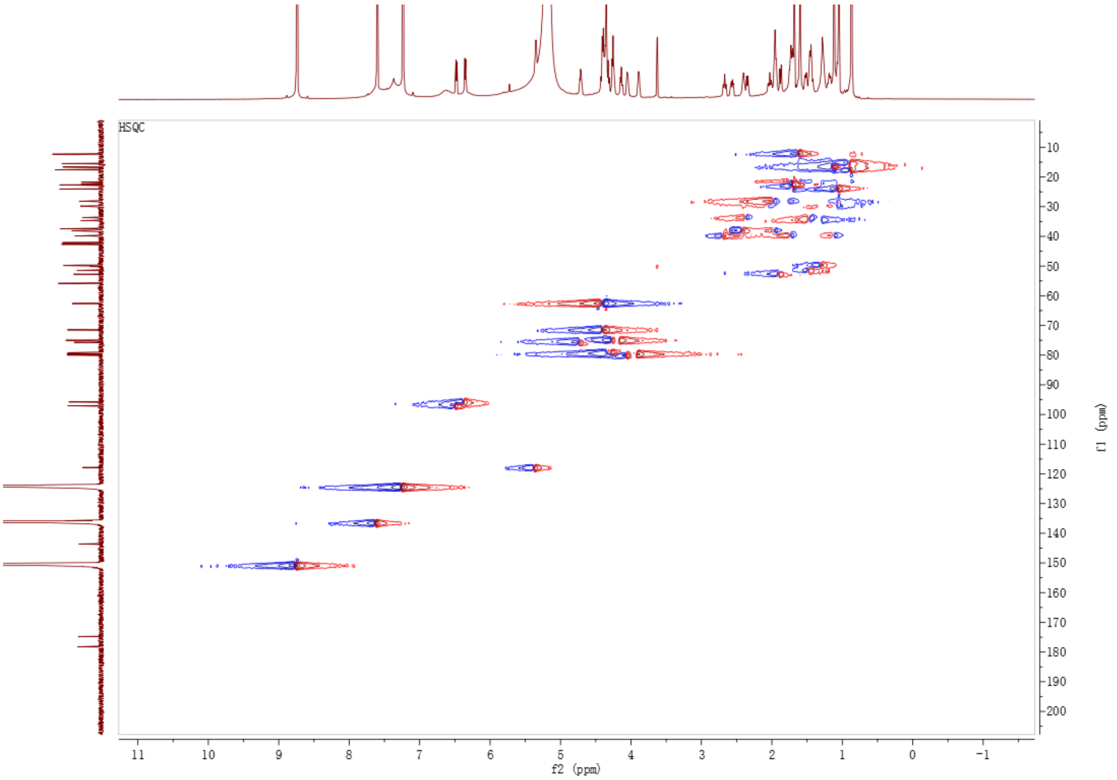

Figure S6. HSQC spectrum of compound **1.**

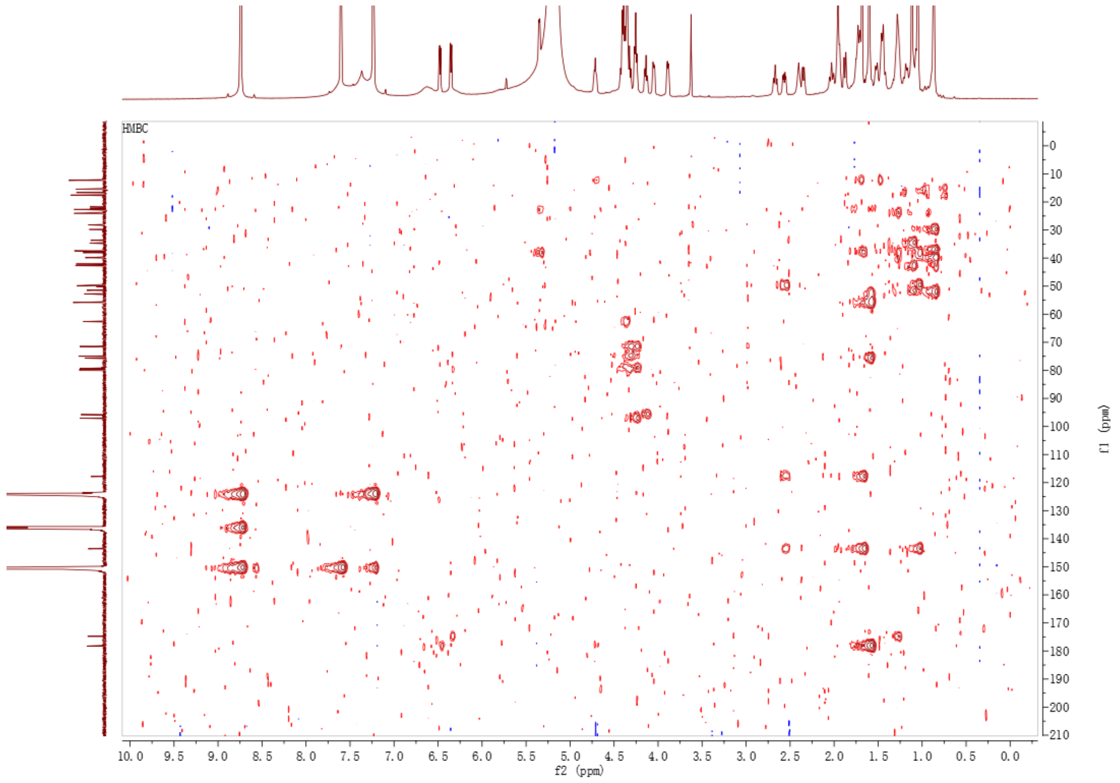

Figure S7. HMBC spectrum of compound **1.**

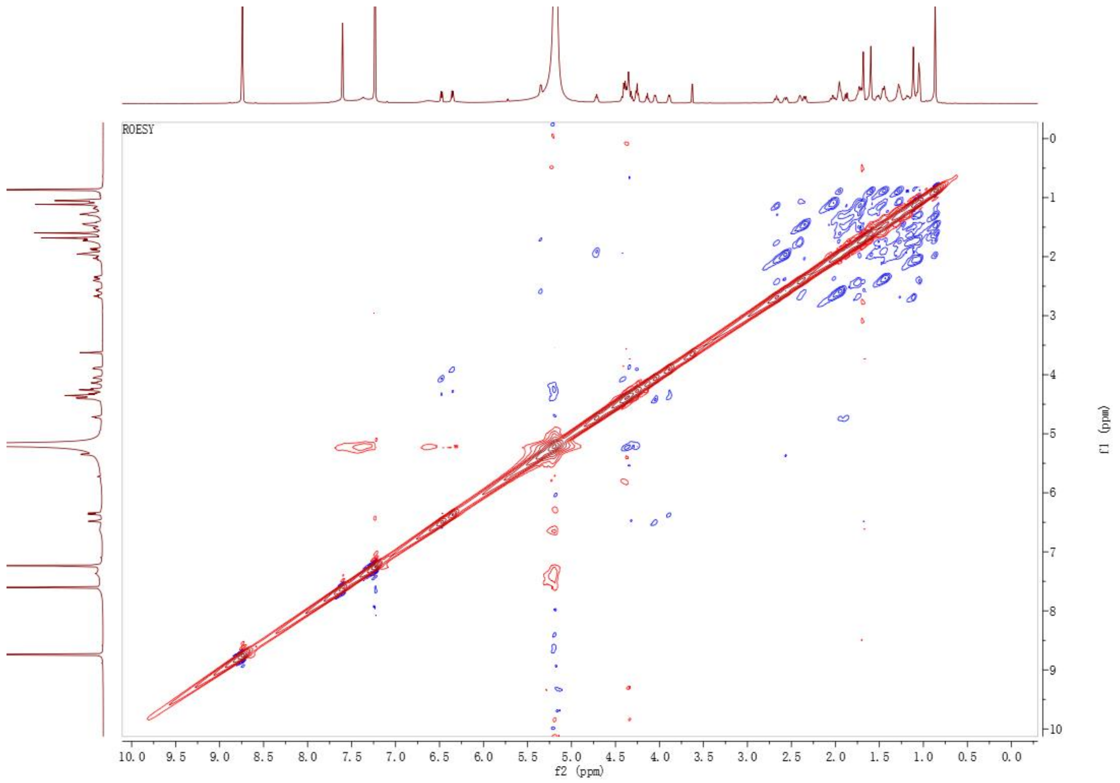

Figure S8. ROESY spectrum of compound **1.**

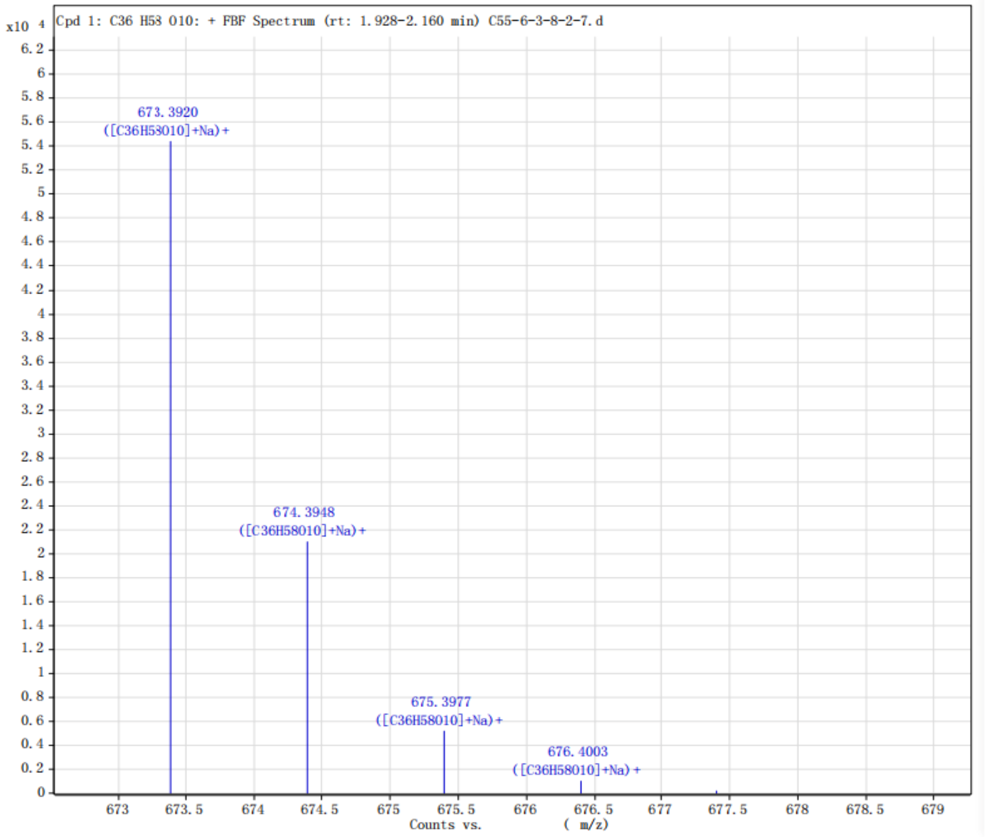

Figure S9. HRESIMS spectrum of compound **2.**

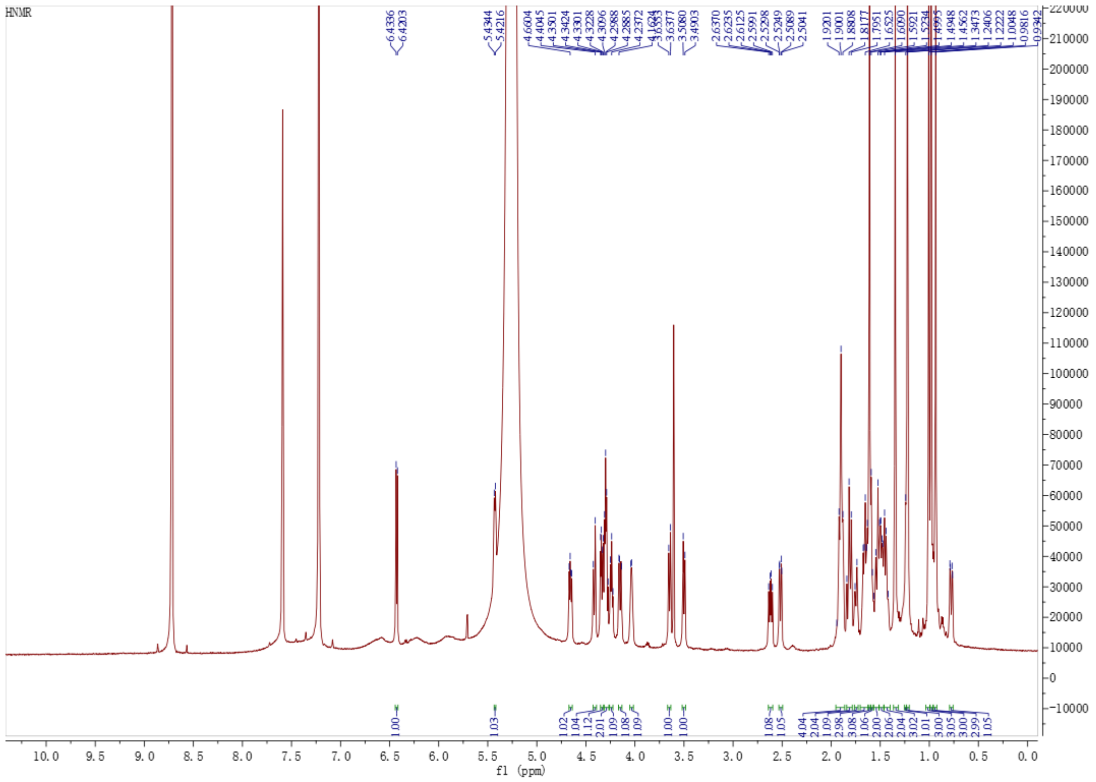

Figure S10. ¹H NMR (600 MHz, Pyridine-d_5) spectrum of compound **2.**

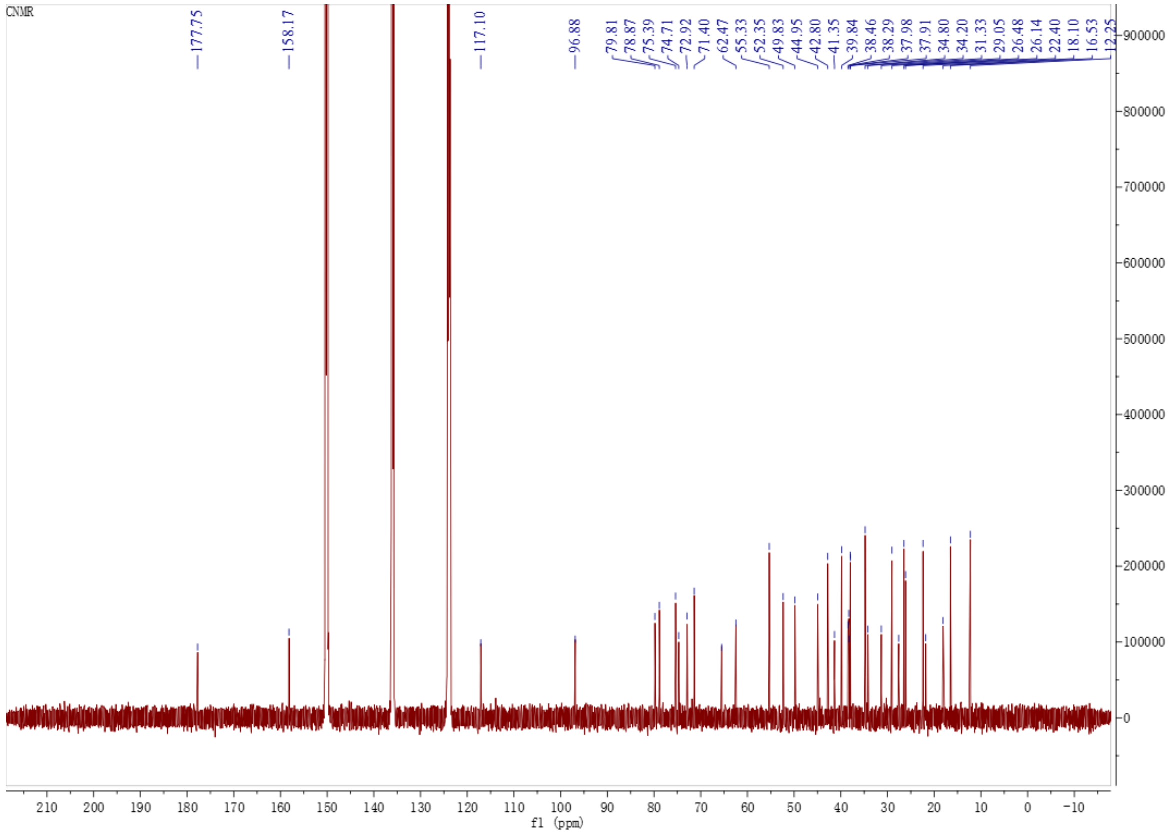

Figure S11. ^{13}C NMR (125 MHz, Pyridine-d_5) spectrum of compound **2.**

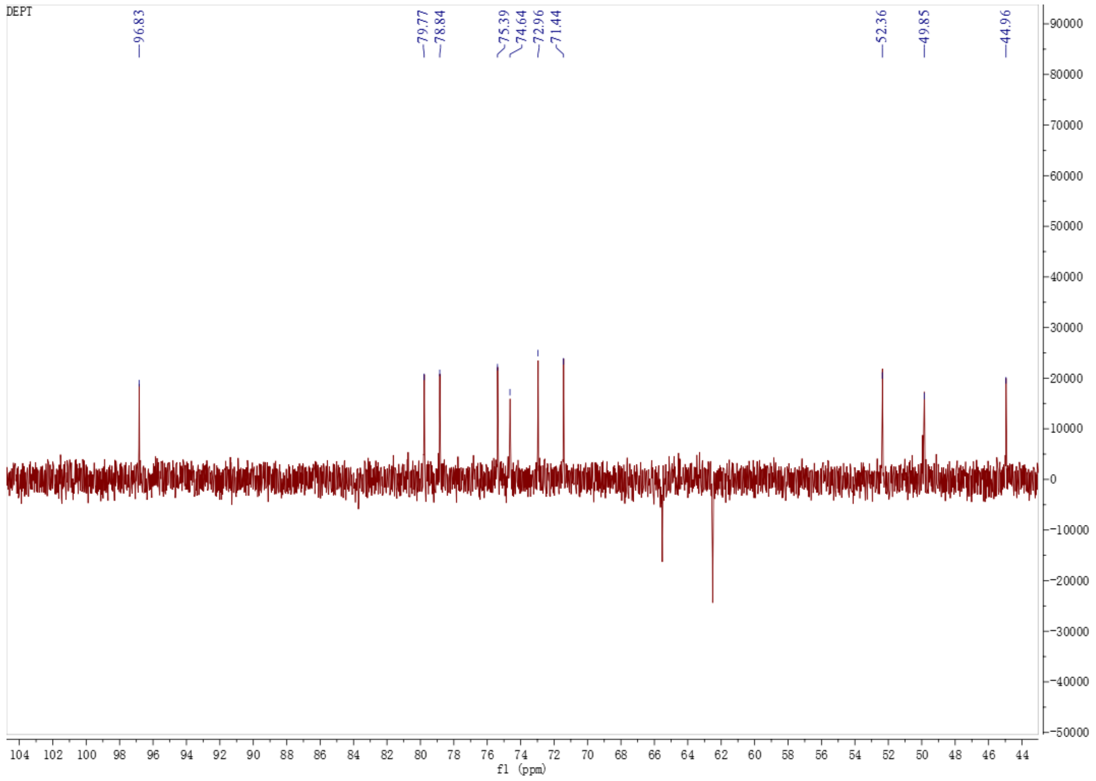

Figure S12. DEPT (125 MHz, Pyridine-d_5) spectrum of compound **2.**

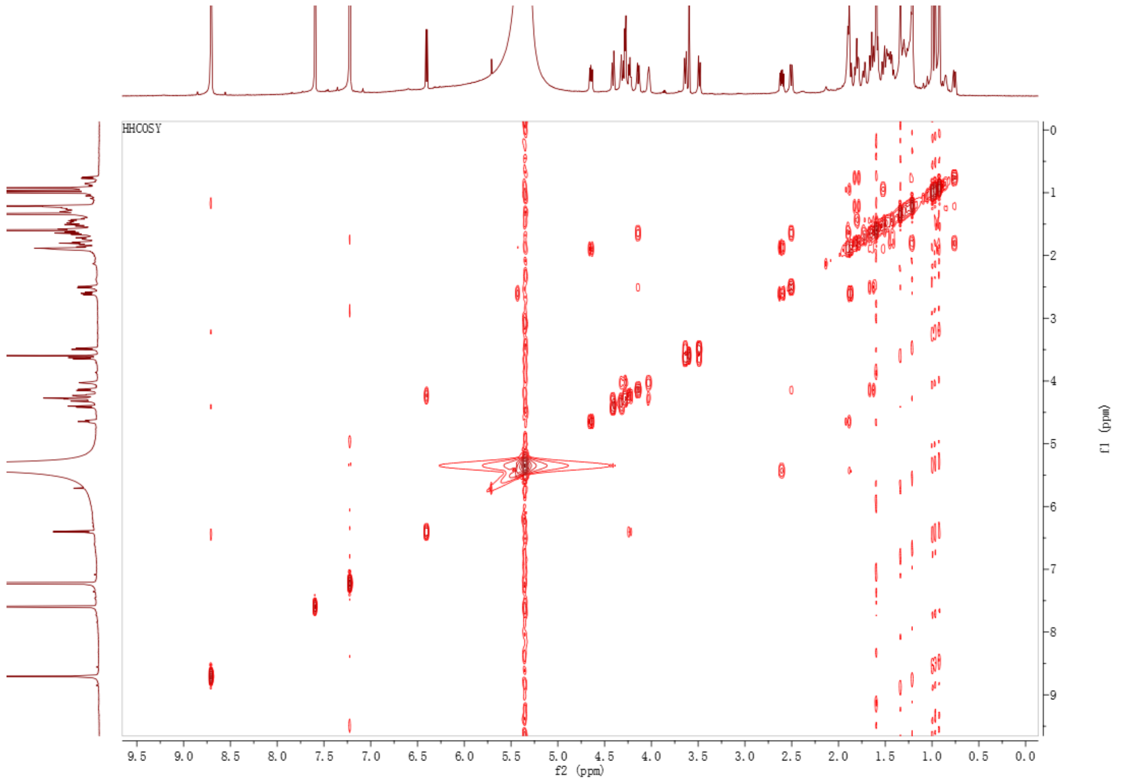

Figure S13. ¹H-¹H COSY (600 MHz, Pyridine-*d*5) spectrum of compound **2.**

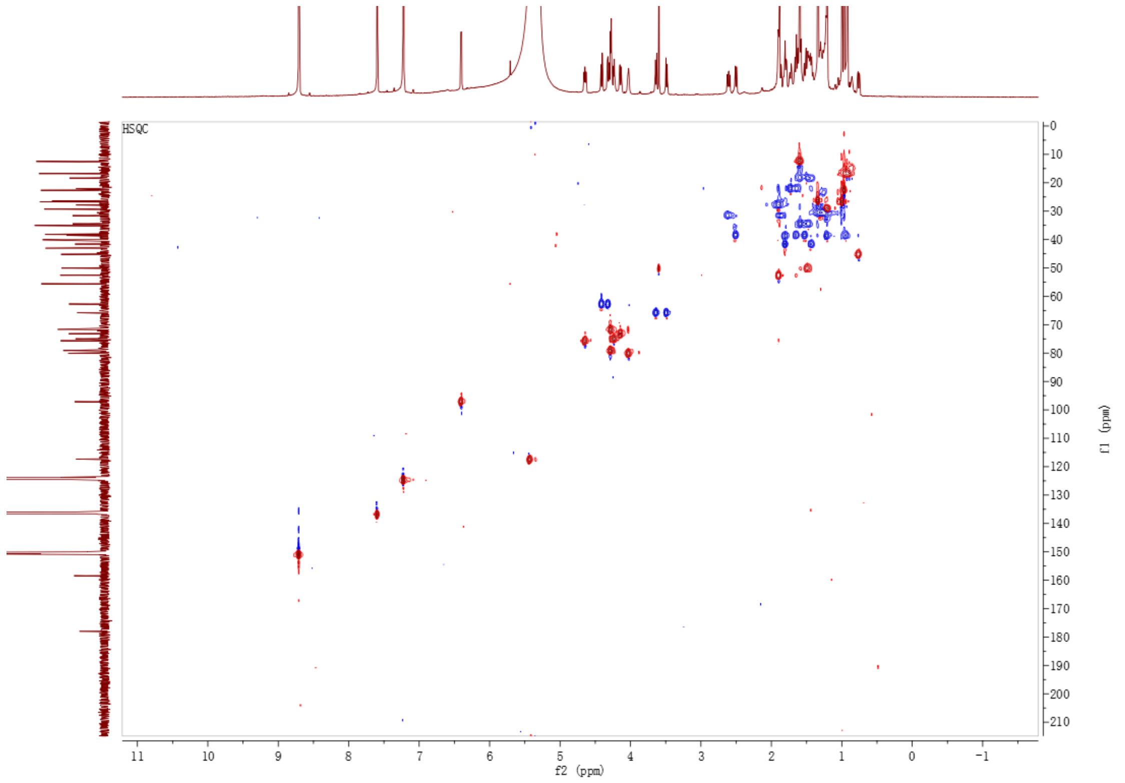

Figure S14. HSQC spectrum of compound **2.**

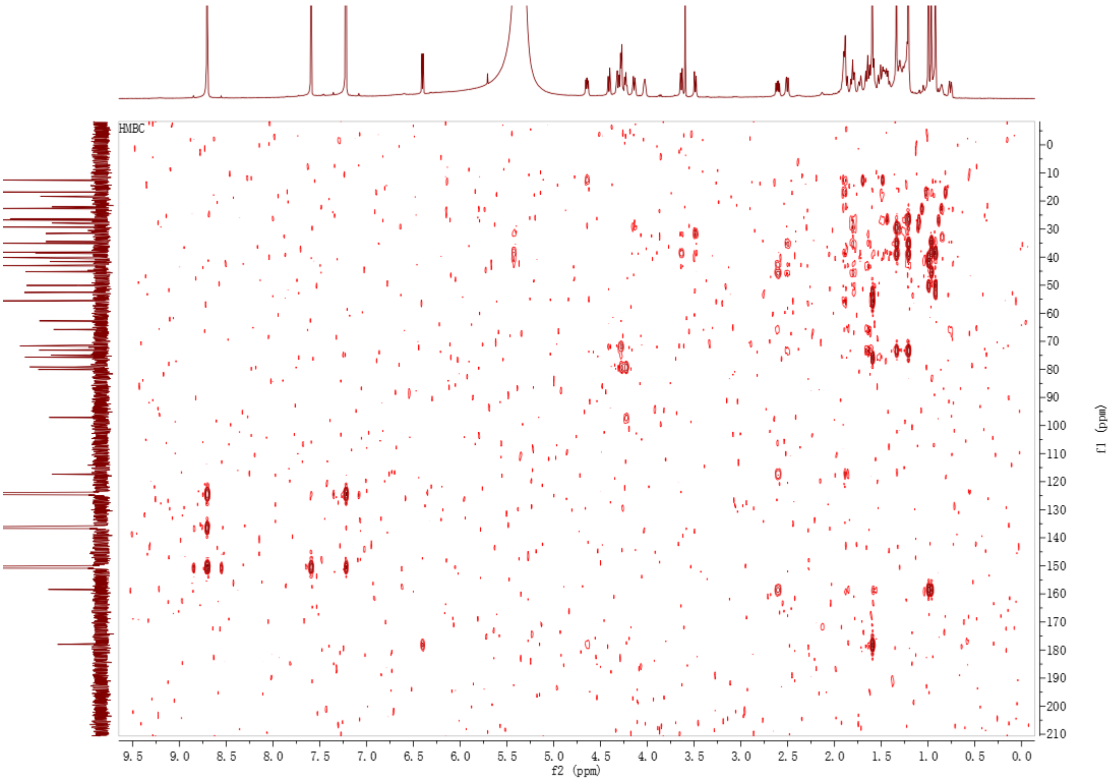

Figure S15. HMBC spectrum of compound **2.**

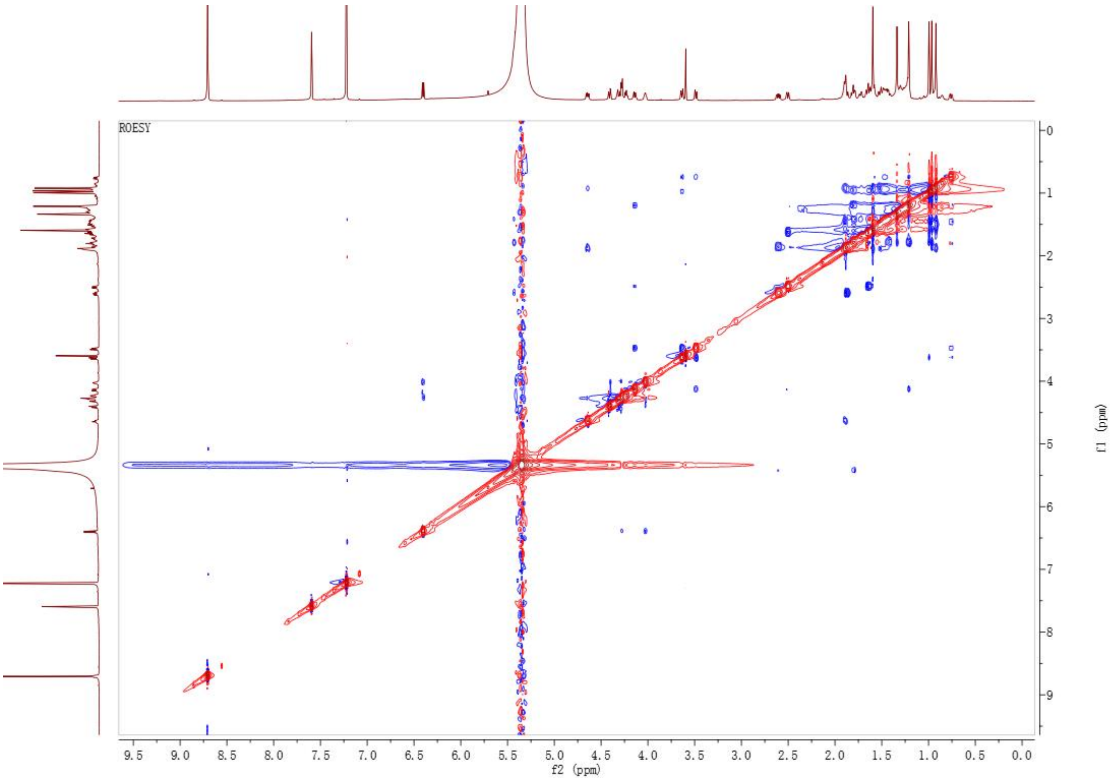

Figure S16. ROESY spectrum of compound **2.**

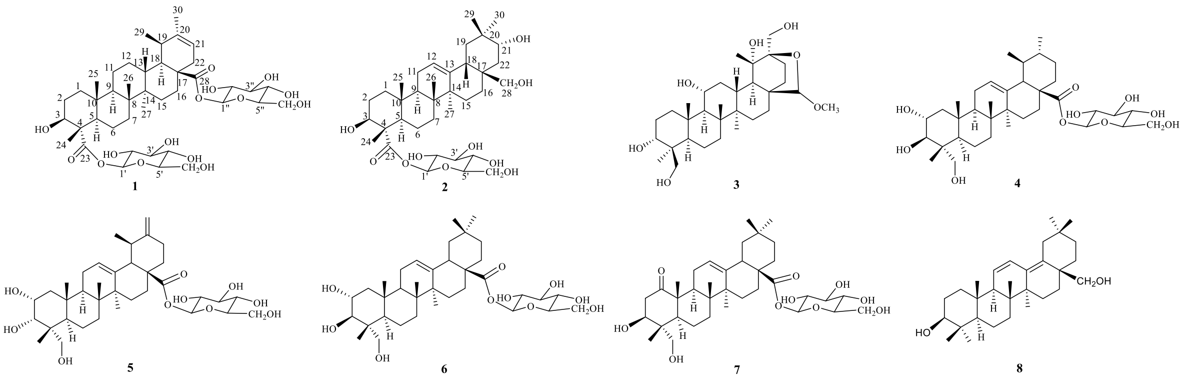

Figure S17. The structures of compounds **1-8** from *H. littoralis.*

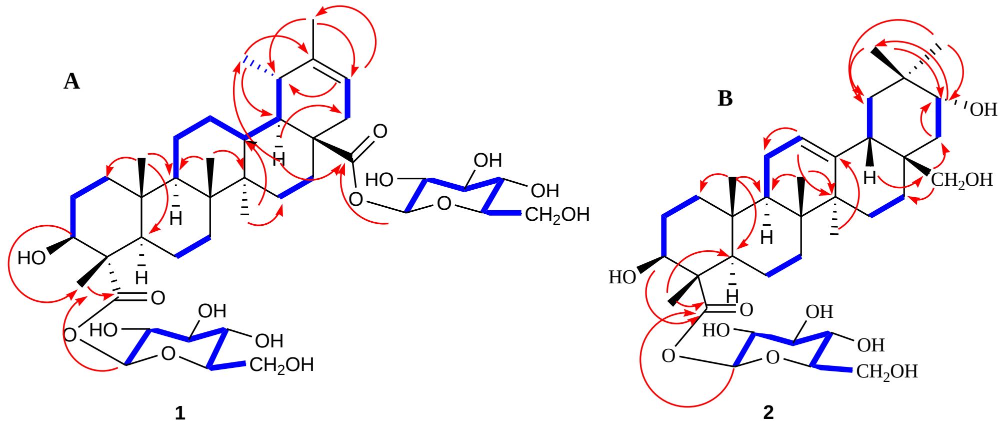

Figure S18. ¹H-¹H COSY (blue bold bonds) and key HMBC (red arrows) correlations of **1** (**A**) and **2** (**B**) .

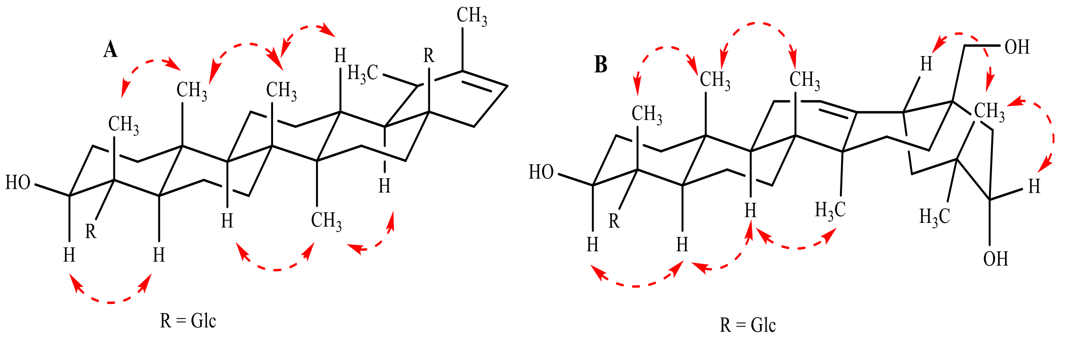

Figure S19. ROESY (double dashed arrows) correlations of **1** (**A**) and **2** (**B**).

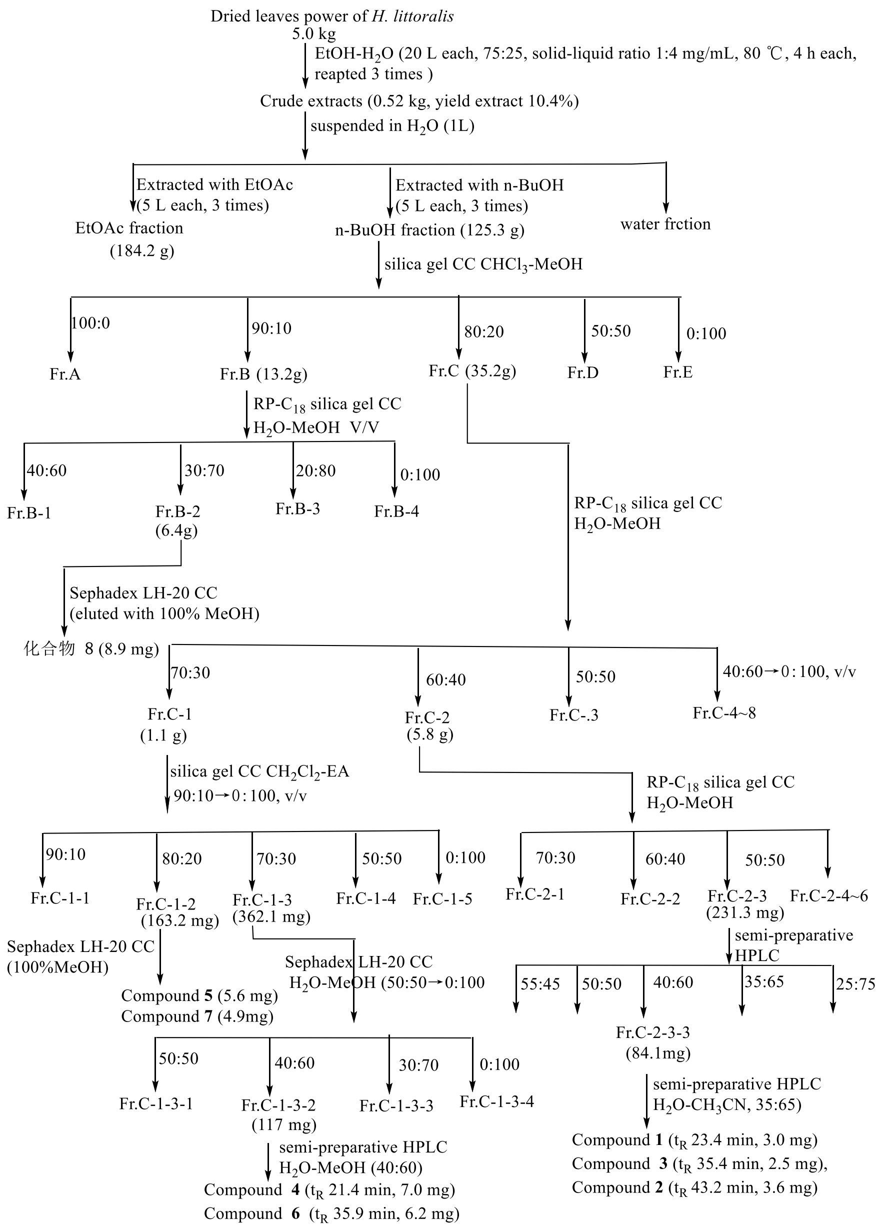

Figure S20. The flowchart for the isolation procedure of *H. littoralis.*
